# Comparative genomic and resistance characterization of ST2 and ST164 carbapenem-resistant Acinetobacter baumannii from hospital environments and clinical specimens

**DOI:** 10.1099/mgen.0.001679

**Published:** 2026-04-08

**Authors:** Xiao Wang, Tongsheng Xu, Yue Zhang, Ye Qiu, Yanru Liang, Jun Feng, Yuanping Wang, Bing Zhao, Lili Ren

**Affiliations:** 1 National Institute of Pathogen Biology, Chinese Academy of Medical Sciences & Peking Union Medical College, Beijing, PR China; 2 Shanghai Pudong New Area Center for Disease Control and Prevention (Shanghai Pudong New Area Health Supervision Institute), Shanghai, PR China; 3 Shanghai Municipal Center for Disease Control and Prevention, Shanghai, PR China

**Keywords:** antimicrobial resistance, carbapenem-resistant *Acinetobacter baumannii*, ST164, ST2, whole-genome sequencing

## Abstract

Carbapenem-resistant *Acinetobacter baumannii* (CRAB) represents a formidable nosocomial pathogen, with healthcare environments acting as critical reservoirs for its dissemination. In this study, we investigated the prevalence, antimicrobial resistance profiles and genomic characteristics of CRAB isolates collected from hospitals in Shanghai, China, between June and December 2024, identifying ST2^Pas^ (84.13%) and ST164^Pas^ (15.08%) as the predominant lineages among the 126 CRAB isolates recovered from clinical (*n*=94), environmental (*n*=29) and healthcare worker (*n*=3) sources. Environmental CRAB accounted for the highest proportions on patient-contact surfaces (34.48%), medical devices (31.03%) and shared items (24.14%). Within the dominant ST2^Pas^ lineage, clinical isolates exhibited higher resistance rates to ampicillin/sulbactam, cefoperazone/sulbactam and levofloxacin, with significantly higher carriage rates of *bla*_TEM-1D_ compared to environmental isolates. Compared to ST164^Pas^, ST2^Pas^ CRAB isolates exhibited greater resistance to amikacin, gentamicin, trimethoprim/sulfamethoxazole and minocycline and a higher prevalence of *aph*(3′)-Ia, *aph*(3″)-Ib, *aph(3′*)-VI, *aph*(6)-Id, *arm*A, *bla*_OXA-66_*, bla*_TEM-1D_, *mph*(E) and *tet*(B), but lower rates of *bla*_CARB-16_, *bla*_NDM-1_ and *bla*_OXA-91_ (*P<*0.05). Notably, comparative genomic analysis suggested putative adaptive differences between the two lineages. ST2^Pas^ retained the T6SS and biofilm-associated genes (*bap*), descriptive genomic features that suggest a potential capacity for active colonization. Conversely, the ST164^Pas^ clone lacked the T6SS gene cluster but was enriched with the surface adhesin *ata* and immune evasion-related genes. Concordantly, ST164^Pas^ CRAB isolates exhibited significantly stronger biofilm-forming capacities than ST2^Pas^ *in vitro*. We hypothesize that these genomic alterations and phenotypic traits may represent a fitness trade-off, potentially conferring a survival advantage under antibiotic pressure. Furthermore, *bla*_OXA-23_ in ST2^Pas^ was predominantly carried on conjugative plasmids restricted to *Acinetobacter* species, whereas *bla*_NDM-1_ in ST164^Pas^ was localized on a broad-host-range non-mobile plasmid, potentially facilitating cross-genus transmission. Although our ST164^Pas^ isolates shared high homology with clinical strains from Zhejiang, China, the genomic localization of *bla*_NDM-1_ differed between the plasmid and chromosome, respectively. These descriptive genomic findings highlight the putative adaptive trajectories of the predominant ST2^Pas^ and emerging ST164^Pas^ clones, underscoring the critical need for comprehensive genomic surveillance, complemented by future phenotypic validation, to monitor their rapid evolution and dissemination.

**Impact Statement**

This study systematically characterized two predominant CRAB lineages, ST2^Pas^ and ST164^Pas^, from Shanghai hospitals. Distinct antimicrobial resistance and descriptive genomic features were identified between environmental and clinical isolates, revealing significant divergence between the classical ST2^Pas^ and emerging ST164^Pas^ lineages. Based on these genomic and in *vitro* phenotypic findings, we hypothesize that ST164^Pas^ may employ a distinct adaptive strategy: despite lacking the T6SS, it appears to compensate through the enrichment of carbapenemase genes (*bla*_NDM-1_, *bla*_CARB-16_ and *bla*_OXA-91_), the KL47 capsular gene cluster and the *ata* adhesin, concordantly with its significantly enhanced biofilm-forming capacity. We propose that these genomic alterations represent a potential fitness trade-off, enhancing its environmental adaptability and survival under severe antibiotic pressure. Furthermore, the localization of *bla*_NDM-1_ on a broad-host-range plasmid suggests a potential capacity for cross-genus transmission. Altogether, these genomic insights highlight ST164^Pas^ as a rapidly evolving high-risk clone, underscoring the urgent need for comprehensive genomic surveillance, complemented by future *in vivo* validation, to monitor its nosocomial dissemination.

## Data Summary

All sequencing data have been submitted to the NCBI and the BioProject number is PRJNA1390075 (https://www.ncbi.nlm.nih.gov/bioproject/PRJNA1390075). Individual accession numbers for all 126 sequences, along with their corresponding genomic quality assessment metrics, are provided in Table S1.

## Introduction

*Acinetobacter baumannii*, a ubiquitous opportunistic pathogen prevalent in soil, water and healthcare settings, represents a leading cause of hospital-acquired pneumonia, bacteraemia, meningitis and wound infections [1]. Characterized by robust environmental persistence, efficient nosocomial transmission and rapid acquisition of antimicrobial resistance (AMR), this organism poses formidable clinical challenges [2]. Notably, *A. baumannii* exhibits exceptional desiccation tolerance and biofilm-forming capabilities, enabling its persistent colonization on hospital surfaces and medical equipment [3]. Over the past decade, the persistent increase in *A. baumannii* infection rates and its accelerated inter-hospital transmission have underscored the severity of cross-contamination between ward environments and patients, identifying the ward environment as a critical reservoir and route of transmission for *A. baumannii*-associated hospital-acquired infections (HAIs) [4]. Carbapenems, once the cornerstone for treating multidrug-resistant Gram-negative infections, have become increasingly ineffective due to the global spread of carbapenemase-encoding genes within *A. baumannii* [5]. Consequently, carbapenem-resistant *A. baumannii* (CRAB) has been designated as a critical priority pathogen by the World Health Organization (WHO) for research and development of novel therapeutics [6]. The substantial disease burden highlights this urgency. It is estimated that CRAB infections result in ~8500 cases and 700 deaths annually in U.S. hospitals [7]. In China, CRAB ranks among the predominant pathogens causing healthcare-associated infections, particularly in intensive care units (ICUs), with national surveillance data showing carbapenem resistance rates persisting above 70% [8,9]. Therefore, sustained surveillance of AMR and molecular epidemiological profiles of CRAB in both healthcare settings and patients, together with elucidation of transmission routes and risk factors, is essential to inform targeted antimicrobial therapy, enhance infection control protocols and prevent further proliferation of resistant lineages.

The resistance mechanism of CRAB primarily involves the production of carbapenemases, with class D OXA-type carbapenem hydrolases (particularly the acquired *bla*_OXA-23_ and intrinsic *bla*_OXA-51_) being key determinants and is partially mediated by class B metallo-*β*-lactamases such as IMP, NDM, SIM and VIM, with a very small proportion mediated by class A KPC and GES *β*-lactamases [10,11]. In China, ST2^Pas^ represents the predominant clone among CRAB isolates, characterized by the carriage of *bla_OXA-23_* and facilitated horizontal gene transfer (HGT) via AbaR-type resistance islands [12]. However, the latest research findings from Zhejiang, China, indicate that ST164^Pas^ may be rapidly emerging as a high-risk strain lineage. ST164^Pas^ CRAB co-harbours *bla*_NDM-1_ and *bla*_OXA-23_ carbapenemase genes, exhibiting significantly higher carbapenem MIC50/MIC90 values than ST2^Pas^ CRAB isolates and conferring enhanced resistance [13]. Notably, ST164 may not be a regionally confined clone. Comparative genomic analysis reveals that it has independently evolved carbapenem resistance in 26 countries across 5 continents, indicating its potential for global spread [13]. Unlike the well-characterized ST2^Pas^ CRAB, ST164^Pas^ CRAB remains sparsely documented in the literature, and comparative analyses of AMR or genomic characteristics between the classic ST2^Pas^ and emerging ST164^Pas^ clones remain limited. Therefore, this study aims to systematically compare the distribution, resistance profiles and carbapenemase expression levels of ST2^Pas^ and ST164^Pas^ CRAB in both hospital environments and patients through antimicrobial susceptibility testing and whole-genome sequencing, to evaluate the prevalence and dissemination risk of ST164^Pas^ CRAB in our region.

## Methods

### Sample collection and isolation

#### Inanimate environment and healthcare worker samples

Ten hospitals within the region were selected as sentinel sites based on geographic location (urban/suburban/peri-urban), patient volume, institutional capacity and collaborative potential. At each hospital, sampling was conducted to capture both inanimate reservoirs and animate vectors. Environmental samples were collected from healthcare facility surfaces (including surgical wards, medical wards, ICUs, etc.), covering six categories: medical instruments, items directly handled by healthcare workers (HCWs), treatment-related items, patient-contact items, cleaning supplies and shared items. Concurrently, biotic samples were collected from HCWs (hand and nasal swabs). During routine hospital operations, a minimum of eight samples per type were collected quarterly from each hospital. From June to December 2024, a total of 1812 samples were collected. All samples were inoculated into TSB medium (Oxoid, UK) and incubated at 37 °C for 48 h. Enriched cultures were then streaked onto CHROMagar Acinetobacter plates (CHROMagar, Shanghai, China) and incubated at 37 °C for 48 h. Presumptive colonies were confirmed as *Acinetobacter* spp. through biochemical testing and identified to species level using MALDI-TOF MS (Bruker, Germany) [14]. While HCWs serve as crucial transmission vectors and temporary reservoirs for CRAB [15], to accurately delineate epidemiological dynamics, isolates recovered from their hands and nasal cavities were explicitly classified as the ‘HCW group’, distinctly separated from the ‘inanimate environment group’.

#### Clinical strains

Ninety-four unique CRAB strains (excluding duplicates from the same patient) were consecutively collected from clinical specimens (sputum, blood, urine, etc.) submitted to the ten participating laboratories during the same period. All strains were confirmed by MALDI-TOF MS and exhibited carbapenem resistance phenotypes, defined as minimum inhibitory concentrations ≥8 µg ml^−1^ for either imipenem or meropenem [16].

### Antimicrobial susceptibility testing

Antimicrobial susceptibility testing (AST) was performed by broth microdilution using a commercial panel for aerobic Gram-negative bacilli (Fosun Diagnostics Technology Co, Shanghai, China). The following 16 agents were tested at the indicated concentration ranges: minocycline (MIN, 1–32 µg ml^−1^), piperacillin/tazobactam (TZP, 4–256 µg ml^−1^), cefoperazone/sulbactam (SCF, 4–256 µg ml^−1^), cefotaxime (CTX, 4–128 µg ml^−1^), ceftazidime (CAZ, 4–128 µg ml^−1^), cefepime (FEP, 4–128 µg ml^−1^), amikacin (AMK, 8–256 µg ml^−1^), gentamicin (GEN, 2–64 µg ml^−1^), ciprofloxacin (CIP, 0.5–16 µg ml^−1^), levofloxacin (LEV, 1–32 µg ml^−1^), imipenem (IPM, 1–32 µg ml^−1^), meropenem (MEM, 1–32 µg ml^−1^), trimethoprim/sulfamethoxazole (SXT, 1–608 µg ml^−1^), tigecycline (TGC, 0.5–16 µg ml^−1^), polymyxin B (PB, 0.25–8 µg ml^−1^) and ampicillin/sulbactam (AMS, 2–64 µg ml^−1^). The *E. coli* ATCC®25922™ strain was used as the control strain for AST. Results were interpreted as susceptible (S), intermediate (I) or resistant (R) according to breakpoints established by the Clinical and Laboratory Standards Institute (CLSI) [16], and CRAB was defined as resistance to either imipenem or meropenem [16,17].

### Whole-genome sequencing, assembly and annotation

*A. baumannii* isolates stored at −80 °C were resuscitated on blood agar plates. Three to five pure colonies were scraped with a sterile loop and transferred to a 1.5 ml microcentrifuge tube containing 500 µl sterile saline to prepare bacterial suspensions. Genomic DNA was extracted using the nucleic acid extraction kit (Tianlong Technology Co., Xi’an, China) based on magnetic bead adsorption, and samples with a concentration ≥10 ng µl^−1^ were subjected to library construction. Whole-genome sequencing was performed on the MGISEQ-T7 platform (MGI Tech Co., Ltd., Shanghai, China) at an approximate depth of 200×. Raw reads were quality-filtered and adapter-trimmed using FastQC (v0.11.9) and Fastp (v0.23.2) software [18]. *De novo* assembly was conducted using SPAdes [19] (v3.15.5) software, with scaffolds generated as the final assembly outputs. Taxonomic classification of the assemblies was performed using Kraken2 (v2.1.2), followed by functional annotation with Prokka (v1.14.16) [20] to ensure downstream analysis focused on the target organism. Sequences were deposited to the NCBI website under the BioProject PRJNA1390075.

### Bioinformatic and phylogenetic analyses

Sequence types (STs) were determined using the PubMLST database (2025) (https://pubmlst.org/). The Pasteur MLST scheme employs seven housekeeping genes (*cpn60*, *fusA*, *gltA*, *pyrG*, *recA*, *rplB* and *rpoB*), whereas the Oxford scheme uses *gltA*, *gyrB*, *gdhB*, *recA*, *cpn60*, *gpi* and *rpoD*. The ABRIcate (v1.0.1) (https://github.com/tseemann/abricate) was used to identify AMR genes and virulence genes in the strains based on the ResFinder and VFDB databases, respectively. Capsular (K locus) and lipopolysaccharide (O locus) typing was performed using Kaptive (v2.1.14) [21]. MOB-Suite (v3.19) [22] was used to identify and reconstruct plasmid sequences from genome assemblies and to predict plasmid conjugative transferability and host range via the MOB-typer module. Core genome alignment was performed using Parsnp (v1.2) [23] to evaluate the phylogenetic relationships among the isolates. For the phylogenetic analysis of the isolates obtained in this study, the *A. baumannii* strain ATCC 19606 (GenBank accession GCF_009035845.1) was employed as the reference genome and served as the outgroup for tree rooting. To provide higher resolution for the global phylogenetic analysis of ST164^Pas^ isolates, the ST164^Pas^ strain MRSN11744 (GenBank accession GCA_016520495.2) was utilized as a specific reference to capture more granular genetic variations. Pairwise SNP distances were subsequently extracted from the core genome alignment using SNP-dists (v0.8.2) [24], and the phylogenetic trees were constructed using maximum likelihood analysis. Visualization was performed via the Chiplot online platform (https://www.chiplot.online). Default parameters were used for all analyses unless otherwise specified.

### Biofilm formation assays

Biofilm formation was assessed via crystal violet (CV) staining. Overnight “Lysogeny Broth cultures” were diluted 1:100 in Lysogeny Broth. Aliquots (200 µl) were inoculated into 96-well plates (with sterile broth as controls) and incubated statically for 24 h at 37 °C. After washing thrice with PBS, biofilms were fixed with absolute methanol (200 µl, 15 min), air-dried and stained with 1% w/v CV (200 µl, 15 min). Following three PBS washes and air-drying, the CV was solubilized in 33% v/v acetic acid (200 µl). The OD_570nm_ was measured. Experiments comprised six technical and three biological replicates [25].

### Ethics considerations

This study was approved by the ethical review board of Shanghai Pudong New Area Center for Disease Control and Prevention (Shanghai Pudong New Area Health Supervision Institute) (record number: PDCDCLL-20250508-005).

### Statistical analysis

SPSS statistical software (v27.0) and GraphPad Prism (v10.3.1) were used for statistical analysis. Categorical variables were presented as frequencies and percentages. The Pearson chi-square test was used for variables with expected cell counts ≥5, while Fisher’s exact test was applied when expected cell counts were <1 or for 2×2 tables with small sample sizes. The Kolmogorov–Smirnov test was utilized to assess the normality of continuous data. For comparisons between two groups, an unpaired t-test was used if it met the normal distribution; if not, the Mann–Whitney test was used. Two-sided and *P*<0.05 was considered to be statistically significant.

## Results

### Characterization of isolates

**Fig. 1.**
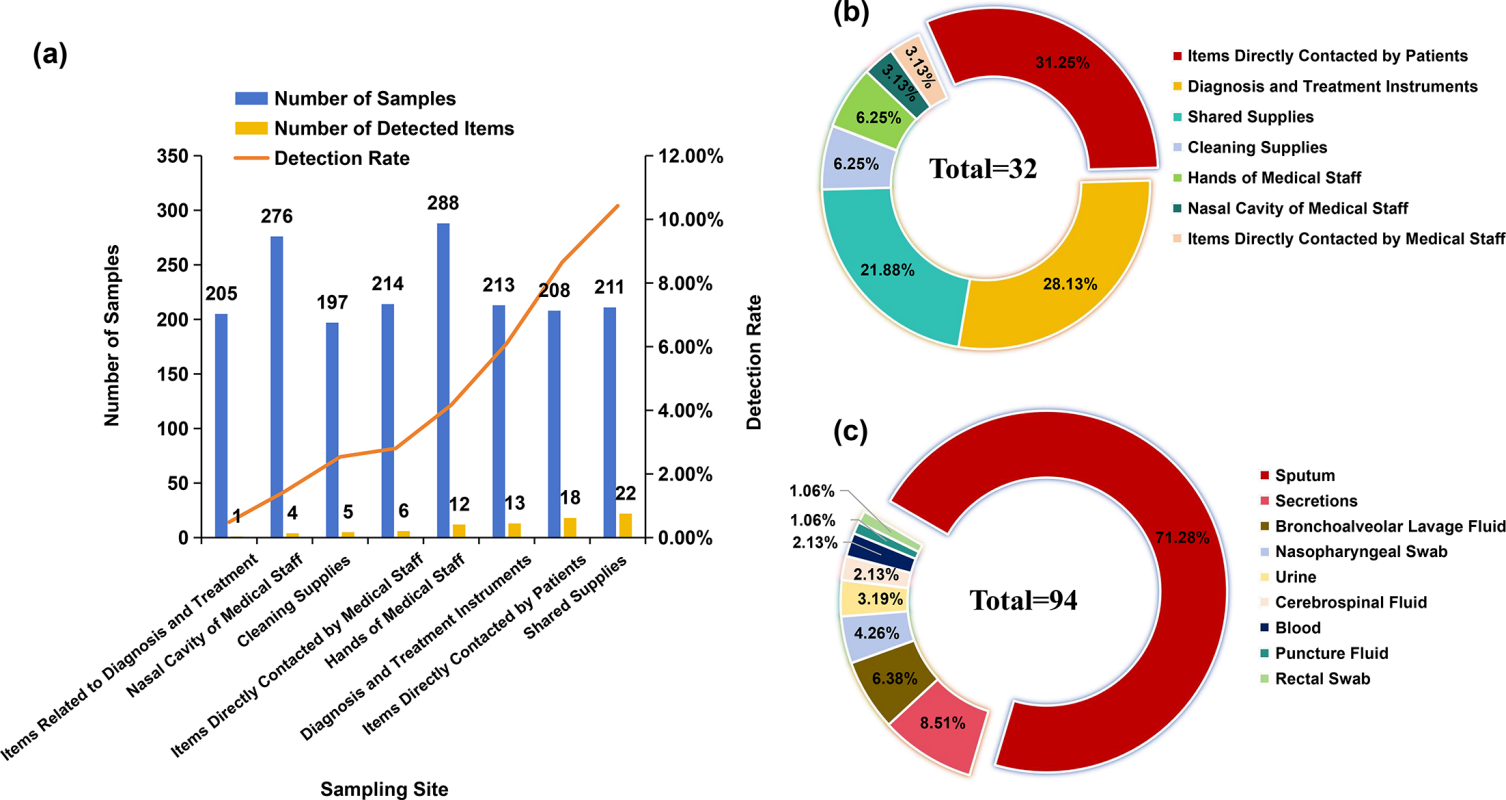
Basic information on CRAB strains. (**a**) Detection of *A. baumannii* on environmental surfaces and HCWs across ten hospitals in Shanghai. (**b**) The distribution of CRAB isolated from hospital environmental surfaces and HCWs. (**c**) The distribution of CRAB isolated from hospital patients. Isolates from ‘secretions’ primarily included wound secretions and pus collected from infected sites, such as pressure ulcers.

From June to December 2024, a total of 1,812 samples were collected from 10 hospitals, comprising 564 HCW samples and 1,248 inanimate hospital environmental samples. Overall, 81 *A*. *baumannii* strains were isolated. Within the inanimate environment, the highest detection rates were observed on shared item surfaces (e.g. door handles and light switches) and patient-contacted surfaces (Fig. 1a). Notably, the detection rate among HCW samples was 2.84% (16/564), with a higher isolation rate from hand swabs (4.17%) than from nasal swabs (1.45%) (Fig. 1a). Among these, CRAB accounted for 39.51% (32/81), predominantly from patient-contacted surfaces (31.25%), diagnosis and treatment instruments (28.13%) and shared items (21.88%) (Fig. 1b).

Concurrently, 94 non-duplicate CRAB clinical isolates were obtained from the same hospitals, primarily from ICU (*n*=41), internal medicine wards (*n*=24), surgical departments (*n*=16), emergency units (*n*=8) and other inpatient wards (*n*=5). These isolates were obtained from various clinical specimens, with sputum representing the majority (71.82%) (Fig. 1c). For genomic analysis, 126 CRAB isolates (including both environmental and clinical strains) underwent whole-genome sequencing, producing assemblies with an average N50 of 152,288 bp (range: 72,340–197,163 bp) and genome sizes ranging from 3.74 to 4.12 Mb (Table S1, available in the online Supplementary Material).

### Sequence typing, capsular polysaccharide locus typing and phylogenetic analysis

**Fig. 2.**
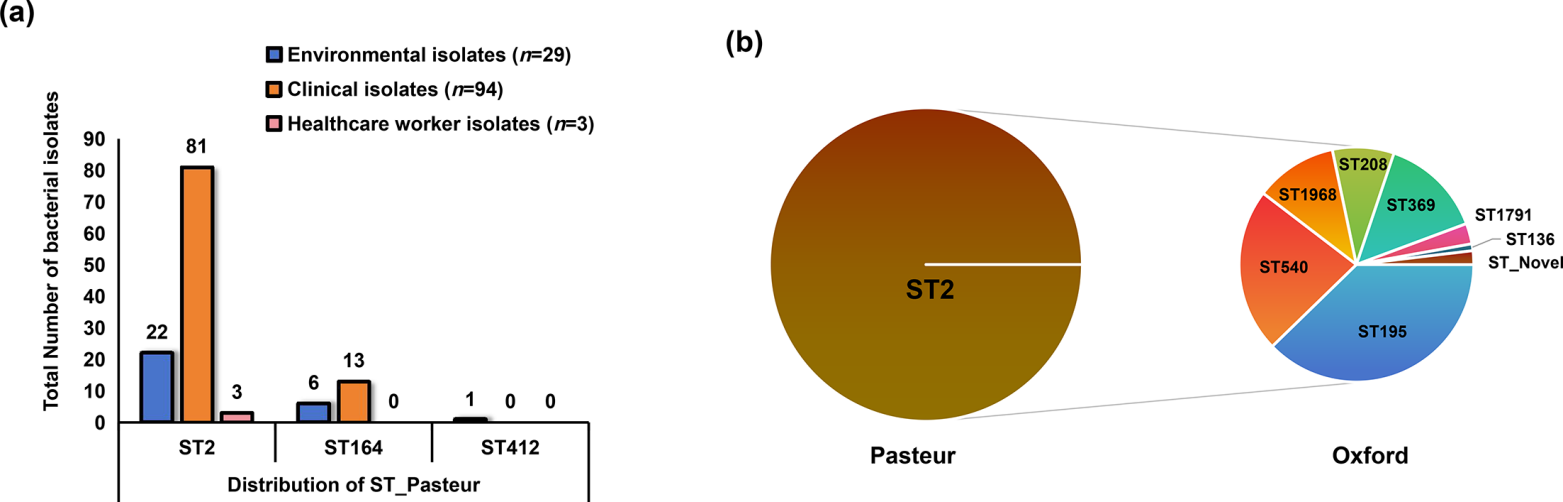
ST distribution of CRAB. (**a**) Distribution of sequence types_Pasteur among CRAB isolates across ten hospitals in Shanghai. (**b**) The compound pie chart shows the subdivision of ST2^Pas^ into Oxford STs.

MLST_Pasteur analysis revealed that the 126 CRAB isolates were grouped into three STs, with ST2^Pas^ predominating (84.13%, 106/126), including 75.86% (22/29) of the environmental isolates, 100% (3/3) of HCW isolates and 86.17% (81/94) of the clinical isolates. ST164^Pas^ accounted for 15.08% (19/126) of isolates, and 1 environmental CRAB was identified as ST412 (Fig. 2a). MLST_Oxford analysis showed that all ST164^Pas^ isolates corresponded to ST1418^Oxf^, whereas ST2^Pas^ isolates were subdivided into nine ST^Oxf^ types (including one novel ST^Oxf^), with ST195^Oxf^ (36.79%, 39/106) and ST540^Oxf^ (23.58%, 25/106) being most prevalent (Fig. 2b). Kaptive identified 11 distinct K-locus variants, dominated by KL3 (30.95%, 39/126) and KL160 (19.84%, 25/126). O-locus typing revealed 3 variants, with OCL1 (84.13%, 106/126) being predominant.

**Fig. 3.**
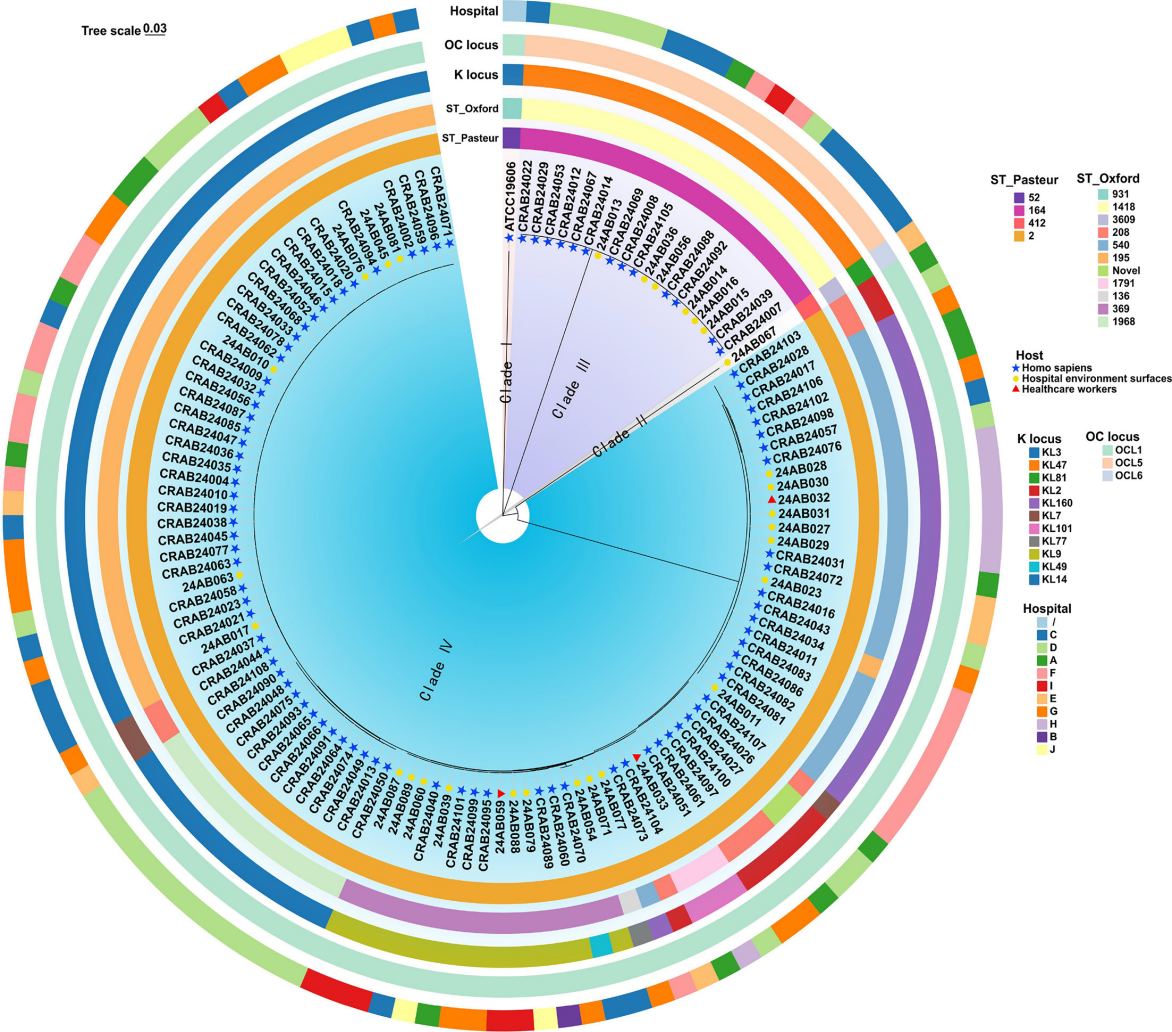
Phylogenetic analysis of 126 CRAB isolates recovered from hospital environments, medical staff and patients across 10 hospitals in Shanghai (June–December 2024). Clade 1, reference strain ATCC 19606 (GenBank accession GCF_009035845.1).

Phylogenetic analysis grouped the 126 CRAB isolates into 3 major clades. Clade 2 comprised ST412^Pas^/ST3609^Oxf^-KL81/OL6 isolates exclusively from hospital environmental surfaces. Clade 3 consisted of ST164^Pas^/ST1418^Oxf^-KL47/OL5 isolates originating from both inanimate hospital environments and patient samples. Meanwhile, clade 4 contained the ST2^Pas^ CRAB isolates, which included the three HCW isolates. Notably, HCW strains 24AB032 and 24AB059 exhibited close evolutionary distances to environmental isolates from the same hospital, whereas HCW strain 24AB033 was genetically closely related to patient isolates from different hospitals (Fig. 3). Statistical analysis revealed no significant difference in ST^Pas^ distribution between environmental and clinical sources (Table S2). However, the prevalence of ST369^Oxf^ among environmental CRAB isolates was significantly higher than that in clinical isolates (Table S3). Due to the limited sample size, isolates obtained from HCW (*n*=3) were excluded from the comparative statistical analysis.

### AMR profiles of ST2^Pas^ and ST164^Pas^ CRAB isolates

All 126 CRAB isolates exhibited 100% resistance to CTX, CAZ, MEM and CIP, while maintaining complete susceptibility to TGC and PB. A total of 23 distinct resistance genes were identified across these isolates, among which the *bla*_OXA-124_ gene was exclusively detected in the ST412^Pas^ CRAB isolate. To further evaluate the AMR profiles between clinical and environmental isolates while controlling for clonal effects, we performed a comparative analysis specifically within the dominant epidemic clone ST2^Pas^ CRAB. The results demonstrated that clinical isolates had significantly higher resistance rates to AMS, SCF and LEV compared to environmental isolates (Table S4). Furthermore, clinical isolates carried significantly higher frequencies of *bla*_TEM-1D_ than environmental isolates (*P*<0.05, Table S5).

Further analysis of AMR profiles in ST2^Pas^ and ST164^Pas^ CRAB revealed that ST2^Pas^ CRAB exhibited significantly lower resistance to FEP but higher resistance to AMK, GEN, SXT and MIN relative to ST164^Pas^ CRAB (*P*<0.05, Table 1). Among ST2^Pas^ CRAB isolates, the carriage rates of *aph*(3′)-Ia, *aph*(3″)-Ib, *aph*(3′)-VI, *aph*(6)-Id, *arm*A, *bla*_OXA-66_, *bla*_TEM-1D_, *mph*(E), *msr*(E) and *tet*(B) were significantly higher than those in ST164^Pas^ CRAB isolates (*P*<0.05, Table 2). In contrast, carriage rates of *bla*_CARB-16_, *bla*_NDM-1_ and *bla*_OXA-91_ were significantly lower in ST2^Pas^ than in ST164^Pas^ CRAB isolates (*P*<0.05, Table 2).

**Table 1.** AMR profiles of ST2 and ST164 CRAB isolates

Category of antibiotics		Antibiotic	ST2 (*n*=106)		ST164 (*n*=19)		*χ* ^2^	*P* value
			Isolate (*n*)	Resistance rate (%)	Isolate (*n*)	Resistance rate (%)		
*β*-Lactams	Penicillins	Piperacillin/tazobactam	105	99.06	19	100.00	–	1.000
		Ampicillin/sulbactam	102	96.23	19	100.00	–	1.000
	Cephalosporins	Cefoperazone/sulbactam	60	56.60	13	68.42	0.926	0.336
		Cefotaxime	106	100.00	19	100.00	/	/
		Ceftazidime	106	100.00	19	100.00	/	/
		Cefepime	66	62.26	18	94.74	7.708	**0.005**
	Carbapenems	Imipenem	104	98.11	19	100.00	–	1.000
		Meropenem	106	100.00	19	100.00	/	/
Aminoglycosides		Amikacin	98	92.45	1	5.26	69.153	**<0.001**
		Gentamicin	104	98.11	0	0.00	104.053	**<0.001**
Quinolone		Ciprofloxacin	106	100.00	19	100.00	/	/
		Levofloxacin	92	86.79	15	78.95	0.294	0.588
Folate inhibitors		Trimethoprim/sulfamethoxazole	66	62.26	0	0.00	25.064	**<0.001**
Tetracycline		Minocycline	29	27.36	0	0.00	5.32	**0.021**
		Tigacycline	1	0.94	0	0.00	–	1.000
Polymyxins		Polymyxin B	1	0.94	0	0.00	–	1.000

–, Fisher’s exact test was used when the expected count in at least one cell was <1, yielding exact *P*-values without chi-square approximation (*χ*2 statistic not reported).

/, Statistical analysis not performed due to identical resistance rates.

Bold value*, P<*0.05.

**Table 2.** Distribution of AMR genes in ST2 and ST164 CRAB isolates

AMR gene	ST2 (*n*=106)		ST164 (*n*=19)		*χ* ^2^	*P* value
	Isolate (n)	Prevalence (%)	Isolate (n)	Prevalence (%)		
*aac*(3)-Ia	6	5.66	0	0.00	–	0.590
*ant*(3″)-Ia	4	3.77	0	0.00	–	1.000
*aph*(3′)-Ia	64	60.38	1	5.26	19.608	**<0.001**
*aph*(3″)-Ib, *aph*(6)-Id	106	100.00	0	0.00	117.362	**<0.001**
*aph*(3′)-VI	0	0.00	8	42.11	40.914	**<0.001**
*arm*A	80	75.47	0	0.00	39.832	**<0.001**
*bla*_ADC-25_, *bla*_OXA-23_	106	100.00	19	100.00	/	/
*bla*_CARB-16_	0	0.00	17	89.47	102.288	**<0.001**
*bla*_NDM-1_	0	0.00	18	94.74	109.755	**<0.001**
*bla*_OXA-66_	106	100.00	0	0.00	117.362	**<0.001**
*bla*_OXA-91_	0	0.00	19	100.00	117.362	**<0.001**
*bla*_TEM-1D_	71	66.98	2	10.53	21.137	**<0.001**
*cat*B8	16	15.09	0	0.00	2.076	0.15
*erm*(C)	5	4.72	2	10.53	0.223	0.637
*mph*(E), *msr*(E)	78	73.58	0	0.00	37.184	**<0.001**
*sul*1	17	16.04	0	0.00	2.294	0.13
*sul*2	32	30.19	0	0.00	6.206	**0.013**
*tet*(B)	105	99.06	0	0.00	110.375	**<0.001**
*tet*(K)	1	0.94	0	0.00	–	1.000

-, – Fisher’s exact test was used when the expected count in at least one cell was <1, yielding exact *P*-values without chi-square approximation (*χ*2 statistic not reported).

/, Statistical analysis not performed due to identical resistance rates.

Bold value*, P<*0.05.

### Comparative analysis of virulence genes and biofilm phenotypes in ST2 and ST164 CRAB isolates

A total of 140 virulence genes across 8 functional categories were identified among the 126 CRAB isolates. Only one immune evasion-related gene exhibited higher carriage in clinical isolates compared to environmental isolates, and the remaining 139 genes showed no significant inter-group differences (Table S6). Notably, comparative analysis between ST2^Pas^ and ST164^Pas^ CRAB isolates revealed distinct virulence gene repertoires. ST2^Pas^ CRAB exhibited significantly higher carriage rates of *pilA*, *bap*, *bauA* and the type VI secretion system (T6SS) associated virulence genes (*clpV/tssH*, *hcp/tssD*, *tagX*, *tssA*, *tssB*, *tssC*, *tssE*, *tssF*, *tssG*, *tssK* and *tssL*) compared to ST164^Pas^ CRAB (*P*<0.05, Table 3). Conversely, the carriage rates of *ata*, *tviB*, *hemO* and multiple immune evasion-related genes were significantly lower in ST2^Pas^ relative to ST164^Pas^ CRAB (*P*<0.05, Table 3).

**Table 3.** Distribution of virulence genes in ST2 and ST164 CRAB isolates

Classification		Virulence gene	ST2 ( *n* = 106 )	ST164 (*n*=19)	*χ* ^2^	*P* value
Adherence		*ompA*	106	19	/	/
		*ata*	0	19	117.362	**<0.001**
		*fimT*, *fimU*, *fimV*, *gspO/pilD*	106	19	/	/
		*hmw1B*, *hmw2B*, *hmw2C*	1	0	–	1.000
		*pilA*	106	0	117.362	**<0.001**
		*pilB*, *pilC*, *pilE*, *pilF*, *pilG*, *pilH*, *pilI*, *pilJ*, *pilM*, *pilN*, *pilO*, *pilP*, *pilQ*, *pilR*, *pilS*, *pilT*, *pilU*, *pilV*, *pilW*, *pilX*, *pilY1*	106	19	/	/
		*tsaP*	105	19	–	1.000
Biofilm formation	AdeFGH efflux pump	*abaR/F/G/H*	106	19	/	/
	Bap	*bap*	105	0	110.375	**<0.001**
	Csu fimbriae	*csuA*, *csuB*, *csuC*, *csuA/B*	102	19	–	1.000
		*csuD*, *csuE*	101	19	–	1.000
	PNAG	*pgaA*, *pgaB*, *pgaC*, *pgaD*, *plc1*, *plc2*, *plcD*	106	19	/	/
Immune evasion	Immune evasion	*lpsB*, *lpxA*, *lpxB*, *lpxC*, *lpxD*, *lpxL*, *lpxM*	106	19	/	/
		*galE*, *galU*	106	19	/	/
		*licB*, *licC*, *licD*	1	0	–	1.000
		*pgi*	106	19	/	/
		*pseB*, *pseC*, *pseF*, *pseG*, *pseH*, *pseI*	9	0	0.700	0.403
		*tviB*	14	19	62.464	**<0.001**
		*ACICU_RS00395*	94	0	63.268	**<0.001**
		*ACICU_RS00400*	94	0	63.268	**<0.001**
		*ACICU_RS00405*	94	0	63.268	**<0.001**
		*ACICU_RS00445*	8	0	0.531	0.466
		*ACICU_RS00450*	8	0	0.531	0.466
		ACICU_RS00455*	33	0	8.037	**0.005**
		*ACICU_RS00460*	8	0	0.531	0.466
		*ACICU_RS00465*	8	0	0.531	0.466
		ACICU_RS00470*	21	0	3.218	0.073
		*ACICU_RS00475*	91	0	59.968	**<0.001**
		*ACICU_RS00485*	106	19	/	/
		*ACICU_RS00500*	106	19	/	/
		*ACICU_RS04565*	106	19	/	/
		*ACICU_RS04570*	80	19	4.490	**0.034**
		*ACICU_RS04575*	80	19	4.490	**0.034**
		*ACICU_RS04580*	81	19	4.224	**0.04**
		*ACICU_RS04585*	81	19	4.224	**0.04**
		*ACICU_RS04590*	81	18	4.224	**0.04**
		*ACICU_RS04595*	80	18	4.490	**0.034**
		*ACICU_RS04605*	80	19	4.490	**0.034**
		*ACICU_RS04610*	106	19	/	/
		*ACICU_RS16840*	106	19	/	/
Iron uptake	Acinetobactin	*barA*, *barB*, *basA*, *basB*, *basC*, *basD*, *basF*, *basG*, *basH*, *basI*, *basJ*	106	19	/	/
		*bauA*	106	0	117.362	**<0.001**
		*bauB*, *bauC*, *bauD*, *bauE*, *bauF*, *entE*	106	19	/	/
	hemO elaster	*hemO*	80	19	4.49	**0.034**
Regulation	Quorum sensing	*abaI*	106	19	/	/
	bfmRS	*bfmR*, *bfmS*	106	19	/	/
Serum resistance	phpG	*pbpG*	106	19	/	/
Secretion system	T6SS	*clpV/tssH*, *hcp/tssD*	95	0	66.123	**<0.001**
		*tagX*	97	0	72.444	**<0.001**
		*tssA*, *tssK*, *tssL*	97	0	72.444	**<0.001**
		*tssB*, *tssC*, *tssE*, *tssG*, *tssM*	95	0	62.133	**<0.001**
		*tssF*	92	0	62.464	**<0.001**
		*vgrG/tssI*	106	19	/	/
	T2SS	*gspC*, *gspD*, *gspE1*, *gspE2*, *gspF*, *gspG*, *gspH*, *gspI*, *gspK*, *gspL*, *gspM*, *gspN*	106	19	/	/

–, Fisher’s exact test was used when the expected count in at least one cell was <1, yielding exact *P*-values without chi-square approximation (*χ*2 statistic not reported).

/, Statistical analysis not performed due to identical resistance rates.

*Bold value*, P<*0.05.

**Fig. 4.**
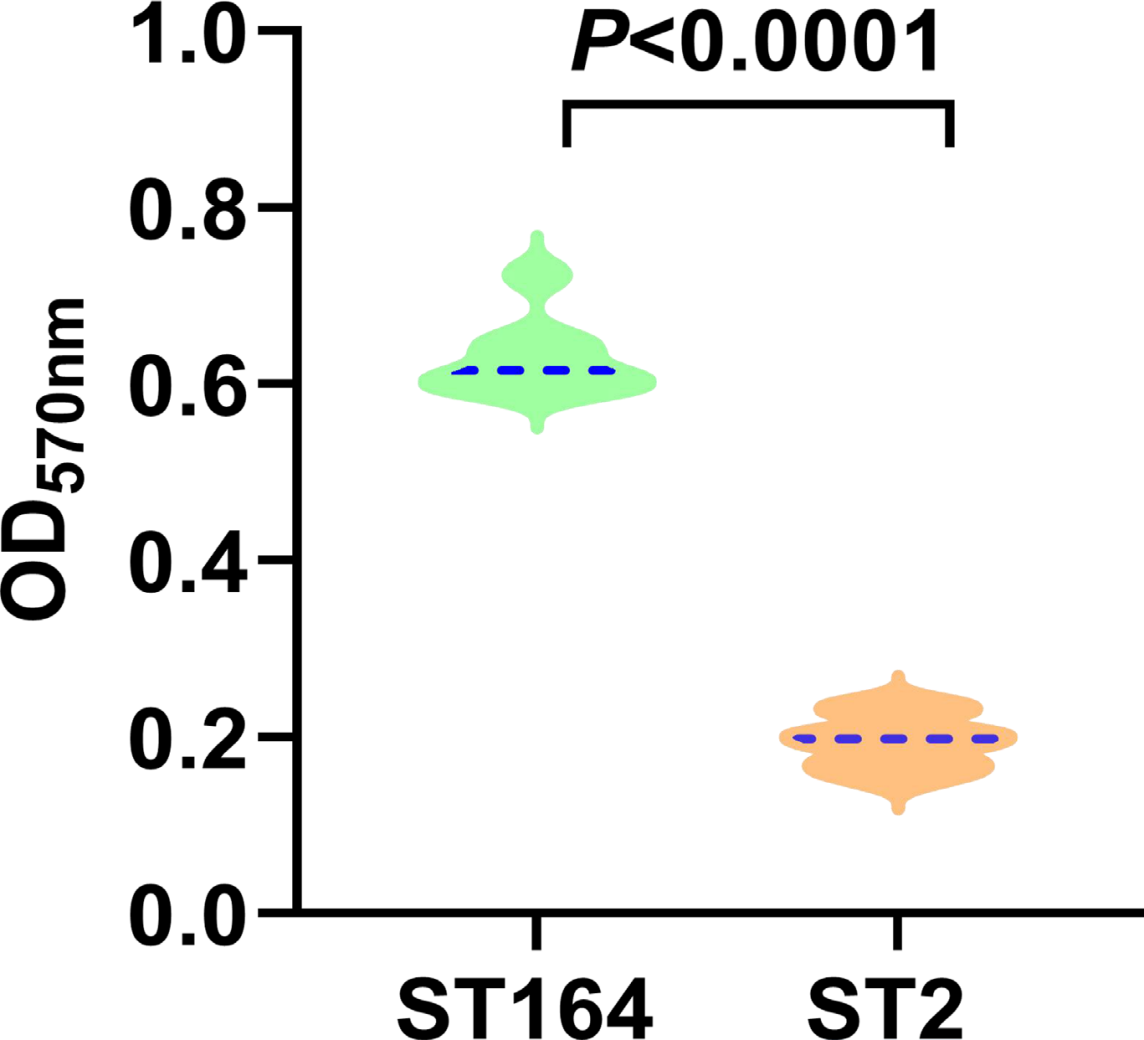
Comparison of biofilm-forming capacities between ST2 and ST164 CRAB isolates.

Although both ST2^Pas^ and ST164^Pas^ CRAB isolates shared a common set of biofilm-associated genetic determinants, including the AdeFGH efflux pump, Csu fimbriae and the *pga* locus (encoding PNAG), they exhibited divergent adhesin repertoires. Specifically, *bap* and *pilA* were more commonly found in ST2^Pas^ CRAB isolates compared to ST164^Pas^ CRAB isolates, whereas the *ata* gene showed a significantly higher prevalence in ST164^Pas^ CRAB isolates. Notably, phenotypic characterization via CV staining revealed that ST164^Pas^ CRAB isolates possessed a significantly enhanced biofilm-forming capacity, producing greater biomass compared to ST2^Pas^ CRAB (*P*<0.0001, Fig. 4).

### Plasmid profiling of ST2 and ST164 CRAB isolates

**Fig. 5.**
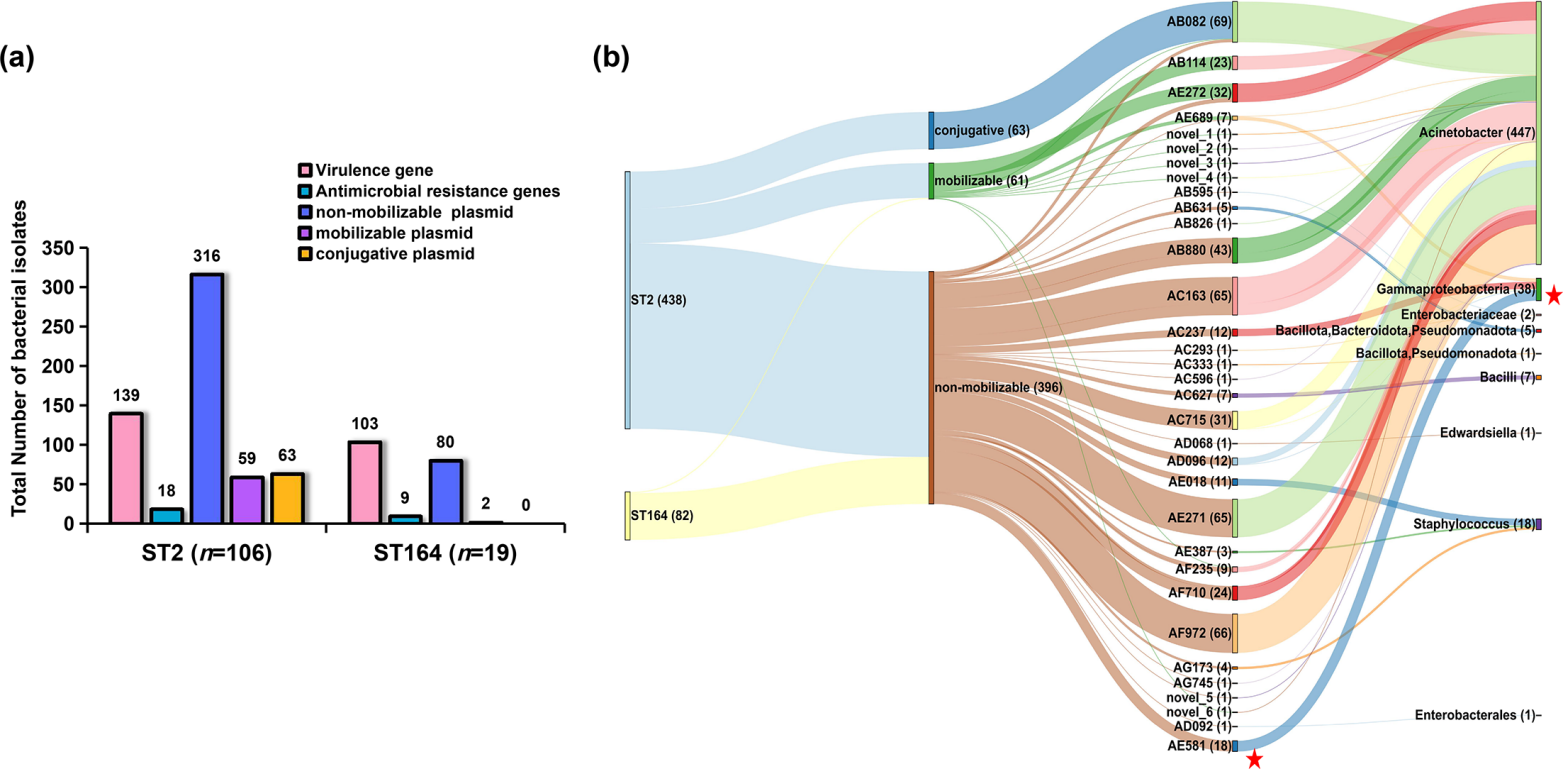
Plasmid profiling of ST2 and ST164 CRAB isolates. (**a**) The bar chart comparing plasmid types and the carriage of resistance and virulence genes between ST2^Pas^ and ST164^Pas^ CRAB. (**b**) The Sankey diagram illustrates the correlation between plasmid types, predicted host ranges and plasmid cluster_id in ST2^Pas^ and ST164^Pas^ CRAB strains. The thickness of each line is proportional to the quantity. Five-pointed star, *bla*_NDM-1_-harbouring plasmid.

MOB-recon module of MOB-Suite (v3.19) identified 438 plasmids among 106 ST2^Pas^ CRAB isolates, of which 14.38% (63/438) were conjugative plasmids harbouring *ISAba26*. Notably, 84.13% (53/63) of these conjugative plasmids carried *bla*_OXA-23_ gene, with predicted hosts restricted to *Acinetobacter* spp*.* (Fig. 5a, Table S7). In contrast, all 82 plasmids from 19 ST164^Pas^ CRAB isolates were non-mobilizable plasmid. All *bla*_NDM-1_ genes in ST164^Pas^ CRAB isolates were localized on the non-mobile plasmid ADE581, with predicted hosts belonging to *Gammaproteobacteria*. Among the 396 predicted non-mobile plasmids, host predictions spanned a broad range, encompassing 9 major host categories including *Acinetobacter* and *Staphylococcus* (Fig. 5b).

**Fig. 6.**
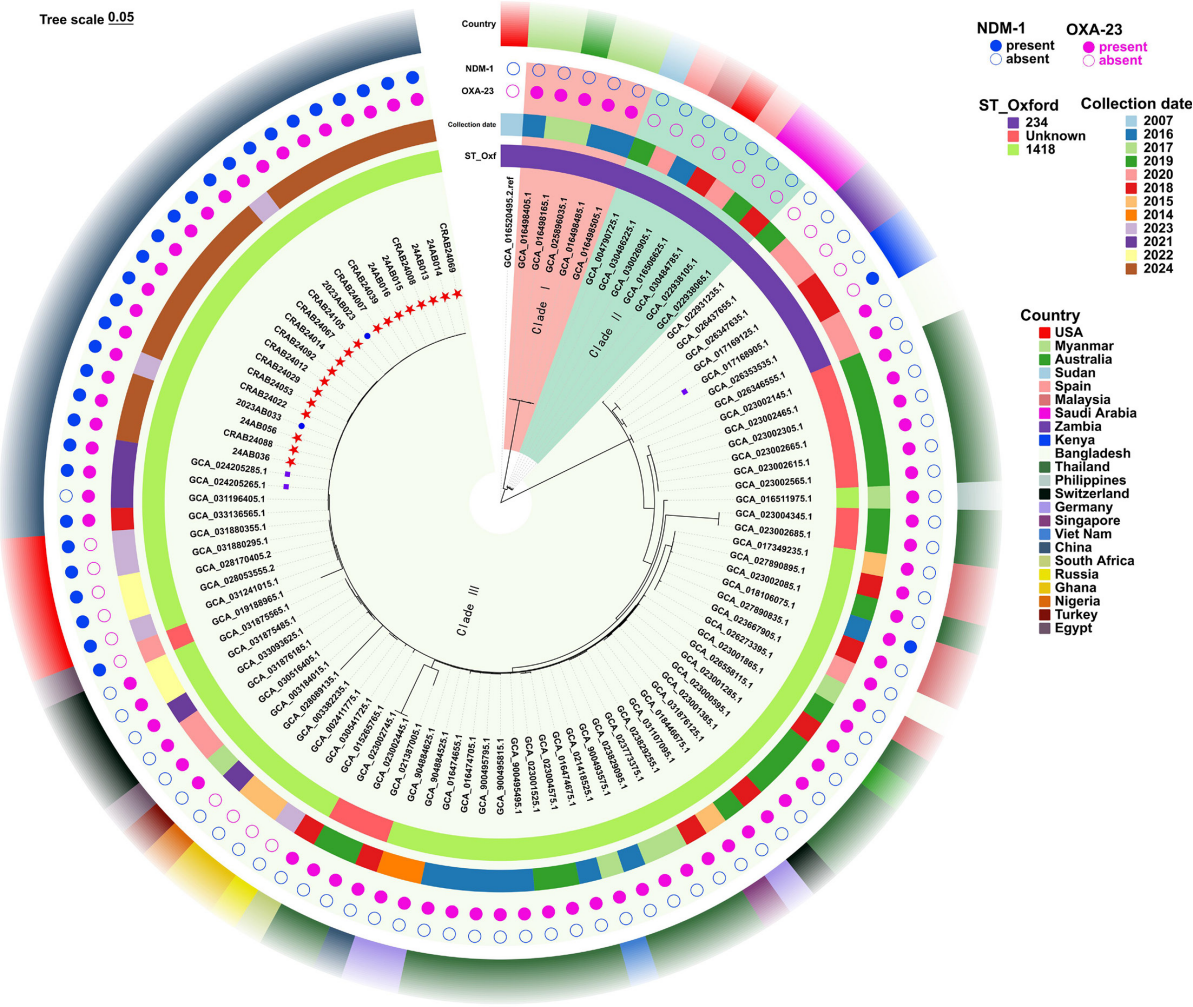
Systematic evolutionary analysis of ST164^Pas^ CRAB from 23 countries. Star, ST164^Pas^ CRAB isolates from this study; circle, ST164^Pas^ CRAB isolates from Shanghai hospital environment in 2023; square, *bla*_NDM-1_ gene located on chromosome. Reference strain, ST164^Pas^ strain MRSN11744 (GenBank accession GCA_016520495.2).

To contextualize the genomic features of ST164^Pas^ globally, 82 ST164^Pas^ *A. baumannii* isolates from 23 countries in the NCBI database were included for comparison (Table S8). Phylogenetic reconstruction revealed two major clonal complexes worldwide: ST164^Pas^/ST234^Oxf^ and ST164^Pas^/ST1418^Oxf^. The ST164^Pas^ isolates obtained in this study from Shanghai exhibited a close phylogenetic relationship with the clinical ST164^Pas^ isolates from Zhejiang reported in 2021, and core genome SNP analysis revealed a narrow genetic distance of only 7–53 SNPs (Fig. 6, Table S9). However, there were critical differences between the Shanghai isolates and the two closest relatives (GCA_024205265.1 and GCA_023001285.1): the *bla*_NDM-1_ gene in the latter two strains was located on the chromosome.

## Discussion

AMR is one of the most critical challenges posing a major threat to global health. Currently, ~700,000 deaths occur annually worldwide due to AMR. It is projected that by 2050, the threat of AMR will surpass that of cancer, making it the leading cause of death globally [26,27]. CRAB categorized by the WHO as a critical priority pathogen, exemplifies this threat [28]. CRAB not only colonizes the human body but also exhibits prolonged persistence on inanimate surfaces, a trait that enhances its environmental fitness [29]. Through HGT, it acquires novel antibiotic resistance genes, thereby facilitating cross-transmission and triggering outbreaks of HAIs, posing a significant threat to global healthcare systems [29]. In this context, we evaluated the contamination status and distribution patterns of CRAB across the inanimate environment and HCW in ten hospitals in Shanghai, China. The results showed that the CRAB detection rates were 2.32% (29/1248) on environmental surfaces, 0.69% (2/288) on the hands of HCW and 0.36% (1/276) in HCW nasal swabs, which were lower than the findings from a 5-year study conducted in Shanghai [30]. Notably, higher CRAB detection rates were observed on items directly contacted by patients (e.g. bedding and pillows), diagnosis and treatment instruments (e.g. ventilators) and shared items (e.g. door handles), identifying these as priority targets for infection prevention interventions. Concurrently, we found that within the dominant ST2^pas^ CRAB lineage, clinical isolates exhibited significantly higher resistance rates to AMS, SCF and LEV compared to environmental isolates. Genotypically, the prevalence of the *bla*_TEM-1D_ gene which confers resistance to *β*-lactam antibiotics, including cephalosporins and piperacillin/tazobactam, was significantly higher in clinical ST2^Pas^ CRAB, and this may further contribute to resistance [31]. These findings underscore the profound impact of therapeutic selective pressure in driving resistance evolution, where clinical isolates under continuous antimicrobial exposure rapidly accumulate resistant phenotypes. In contrast, environmental CRAB isolates lack such selection pressure, resulting in relatively lower levels of drug resistance. Furthermore, the phylogenetic analysis to some extent reveals the complex origins of the HCW isolates. The close genetic relatedness between HCW strains 24AB032 and 24AB059 and the environmental isolates from the same hospital suggests that these cases might represent occupational exposure; that is, HCWs potentially acquire a transient carriage state through contact with contaminated inanimate surfaces during their daily routines. Conversely, the clustering of HCW strain 24AB033 with clinical patient isolates from different hospitals indicates the possibility of alternative acquisition pathways, such as direct patient contact or the regional spread of a highly conserved ST2^Pas^ sub-lineage across different medical institutions. In summary, these findings imply that HCW may be at the critical intersection of the patient-to-person and environment-to-person transmission networks [32], emphasizing the necessity of strict hand and nasal hygiene and environmental disinfection to break these potential transmission cycles.

CRAB has been widely reported and poses a global threat. ST2^Pas^ CRAB, belonging to global clone 2, is generally recognized as a high-risk clonal lineage associated with multidrug resistance and global dissemination [33]. Recent evidence suggests that ST2^Pas^ CRAB in ICUs may be evolving toward the ST164^Pas^ clonal lineage [13]. And ST164^Pas^ CRAB strains can significantly enhance their resistance to ultraviolet radiation by synthesizing pyomelanin and exhibit strong genomic plasticity and environmental adaptability [34]. Furthermore, the emerging high-risk ST164^Pas^ clone can simultaneously carry both the *bla*_NDM-1_ and *bla_OXA-23_* carbapenemase genes, conferring higher resistance to carbapenem antibiotics, and has presumably spread widely across Asia, particularly in China and Thailand [13]. Against this backdrop, our data indicate that ST2^Pas^ remains the predominant clonal lineage for CRAB in Shanghai, but ST164^Pas^ has emerged as a new high-risk clone that warrants attention. Importantly, ST164^Pas^ CRAB was concurrently detected on inanimate environmental surfaces across different departments of multiple hospitals and in patient samples. Moreover, the environmental detection rate of ST164^Pas^ CRAB showed an upward trend, increasing from 0.14% (2/1476) in 2023 to 0.33% (6/1812) in 2024 [14], aligning with concerns raised regarding ST164^Pas^ clonal succession [13] and underscoring its escalating transmission potential. Reassuringly, within the surveillance scope of this study, no ST164^Pas^ CRAB isolates were detected in samples from HCW, suggesting that this clone might not have yet established colonization or transmission among healthcare personnel. Further analysis revealed that all ST164^Pas^ CRAB isolates detected in this study belonged to the ST1418^Oxf^-KL47/OL5 lineage. The KL47 capsular gene cluster is involved in the synthesis, processing and glycosylation of capsular polysaccharides, playing a crucial role in immune evasion and resisting environmental stresses such as antibiotics, disinfectants and desiccation [35]. This mechanism may serve as a contributing factor to the persistent survival and potential nosocomial transmission capacity of ST164^Pas^ CRAB in healthcare settings.

Resistance genes and virulence genes facilitate the adaptive evolution and dissemination of *A. baumannii* under adverse conditions [36]. Analysis of the AMR profiles and genomic characteristics of ST2^Pas^ and ST164^Pas^ CRAB revealed significant functional divergence in their resistance and virulence mechanisms. ST2^Pas^ CRAB isolates universally carried aminoglycoside-modifying enzyme genes [*aph*(3′)-Ia, *aph*(6)-Id and *arm*A], macrolide resistance genes [*mph*(E), *msr*(E)] and tetracycline resistance determinants [*tet*(B)] [31], predicted to confer a multi-target, broad-spectrum resistance phenotype consistent with established ST2^Pas^ CRAB profiles [37]. In contrast, ST164^Pas^ CRAB isolates were significantly enriched in carbapenemase genes (*bla*_NDM-1_, *bla*_CARB-16_ and *bla*_OXA-91_) and demonstrated potent carbapenem hydrolysis activity, consistent with observations from Zhejiang Province, China [13]. This distinct resistance profile raises the possibility that ST164^Pas^ may have evolved adaptively under the selective pressure of carbapenem antibiotics. Additionally, ST164^Pas^ CRAB exhibited high susceptibility to AMK, GEN, SXT and MIN, providing a potential option for clinical combination therapy. However, caution is warranted regarding the risk that ST164^Pas^ may acquire resistance genes (e.g. those conferring aminoglycoside resistance) through HGT during treatment.

Virulence profiling revealed that the ST2^Pas^ CRAB isolates harboured a complete T6SS (*clpV/tssH*, *hcp/tssD* and *tssA-L*), which is associated with the virulence of *A. baumannii* and may potentially enhance the pathogenicity of the carrier strains [38]. Additionally, ST2^Pas^ CRAB isolates carried the critical biofilm-associated gene *bap* [39] and key adhesion factors (*pilA, bauA*). It is hypothesized that the fimbriae, biofilm-associated proteins and iron transport systems encoded by these genes might cooperatively facilitate active host colonization and invasion, potentially assisting the bacteria in evading host immune responses to establish infection [36,40]. In contrast, the ST164^Pas^ CRAB isolates entirely lacked the T6SS gene cluster. Literature suggests that T6SS-deficient strains may paradoxically exhibit enhanced biofilm-forming capabilities [41]. Concordantly, biofilm assays demonstrated that the ST164^Pas^ CRAB isolates possessed significantly stronger biofilm-forming ability than ST2^Pas^, which might be attributed to a fitness cost trade-off during bacterial evolution and the absence of T6SS-mediated interbacterial competition within the biofilms [41]. Furthermore, the ST164^Pas^ CRAB isolates were enriched with the surface adherence factor *ata* [42] and immune evasion-related genes. Considering reports that the overexpression of adhesion genes (such as *bap*) under low-iron conditions can influence biofilm formation [43], these genomic features suggest the possibility that the ST164^Pas^ clone possesses a superior capacity for persistent survival and colonization on inanimate environmental surfaces, such as medical devices.

From a bacterial perspective, conjugative plasmids serve as ideal vehicles for interspecies transfer of resistance genes. Fortunately, only a limited number of *Acinetobacter* plasmids possess conjugative properties capable of transferring resistance genes to new hosts [44]. In our study, half of the ST2^Pas^ CRAB isolates carried conjugative plasmids harbouring *bla*_OXA-23_ gene, with the *ISAba26* insertion element further facilitating horizontal transfer of *bla*_OXA-23_ between plasmids. Host prediction confined these plasmids to the *Acinetobacter* genus, suggesting that ST2 CRAB primarily disseminates carbapenemase genes through intra-species HGT. In contrast, ST164^Pas^ CRAB lacked conjugative plasmids, with the *bla*_NDM-1_ gene stably localized on the non-mobilizable plasmid ADE581, consistent with earlier findings [45]. Nevertheless, the *bla*_NDM-1_ gene can still be mobilized to plasmids or chromosomes of adjacent hosts via helper plasmid [45]. Predicted hosts for the ADE581 plasmid in ST164^Pas^ CRAB extend across the entire *Gammaproteobacteria* class, suggesting that this plasmid likely originated from a transgeneric horizontal transfer event. Notably, the overlapping distribution of ST164^Pas^ CRAB isolates from hospital environments and patients in our study indicates potential cross-transmission between environmental and patient sources. Additionally, although the ST164^Pas^ CRAB isolates in this study were phylogenetically highly close to those from Zhejiang Province, the *bla*_NDM-1_ gene was located on the chromosome in the Zhejiang isolates but on a plasmid in the isolates of Shanghai, China. This discrepancy suggests that the ST164^Pas^ clonal strain may have undergone complex genetic recombination events or adaptive selection during its evolution, implying that ST164^Pas^ CRAB may be undergoing a phase of rapid adaptive evolution.

Taken together, we hypothesize that the ascendancy of ST164^Pas^ CRAB represents an adaptive evolutionary event driven by carbapenem-selective pressure. Genomically, ST164^Pas^ CRAB isolates were enriched with dual carbapenemase genes (*bla*_NDM-1_, *bla*_OXA-23_) and the broad-host-range ADE581 plasmid, which are predicted to confer distinct survival and dissemination advantages in clinical settings [13,34, 46]. Furthermore, the absence of the energy-intensive T6SS [47,48] combined with enriched surface adhesin *ata* and immune evasion-related genes might theoretically represent a fitness trade-off, minimizing energetic expenditure while potentially enhancing long-term persistence on inanimate surfaces. However, as these survival strategies are inferred solely from genomic data, future phenotypic and *in vivo* studies are warranted to substantiate these hypotheses.

This study has several limitations. First, functional validation experiments to confirm the conjugative transfer capability of the plasmid were not conducted. Second, the complete plasmid structures remain unresolved due to the lack of long-read sequencing, and the dynamic integration process of *bla*_NDM-1_ from plasmid to chromosome was not tracked through experimental evolution. Third, the limited clinical metadata precluded comprehensive correlation analysis between strain-specific genomic features and clinical prognostic indicators. Future work will prioritize addressing these gaps through integrated genomic-clinical investigations.

## Conclusions

This study depicts the epidemiological distribution of CRAB in Shanghai hospitals, revealing that ST2^Pas^ was the predominant lineage, while ST164^Pas^ has emerged as a potential high-risk lineage. Patient-contacted surfaces, shared items and medical instruments were the primary areas of CRAB contamination. Within the predominant ST2^Pas^ lineage, clinical isolates exhibited higher resistance to AMS, SCF and LEV compared to environmental isolates, and this phenotypic difference was believed to be related to persistent antibiotic selection pressure. Comparative genomic analysis revealed different resistance profiles and possible adaptive characteristics: ST2^Pas^ CRAB isolates carried a wide range of resistance determinants (such as *aph*, *arm*A and *tet*), as well as a complete T6SS and biofilm-associated genes, indicating that they may have the ability to actively colonize and invade the host. In contrast, ST164^Pas^ CRAB isolates were enriched with carbapenemase genes (*bla*_NDM-1_ and *bla*_OXA-91_). Despite lacking T6SS-related genes, they possessed the KL47 capsule gene cluster and the *ata* adhesin and exhibited stronger biofilm formation ability, which theoretically may compensate for this deficiency to enhance their environmental adaptability. Additionally, the broad-host-range plasmid carrying *bla*_NDM-1_ indicates the theoretical possibility of cross-genus transmission within the *Gammaproteobacteria* class. In conclusion, these descriptive genomic associations suggest that ST164^Pas^ CRAB may be undergoing rapid adaptive evolution, thus requiring close monitoring. However, since these survival and pathogenic strategies are entirely inferred based on genomic data, future phenotypic assays and *in vivo* models are needed to validate these hypotheses.

## Supplementary material

10.1099/mgen.0.001679 Uncited Supplementary Material 1.

## Abbreviations

AST: antimicrobial susceptibility testing
CRAB: carbapenem-resistant *Acinetobacter baumannii*
CV: crystal violet
HAI: hospital-acquired infection
HCW: healthcare worker
HGT: horizontal gene transfer
ICUs: intensive care units
ST: sequence type
T6SS: type VI secretion system
WHO: World Health Organization
